# Application of chlorous acid water for disinfection of surgical site in dairy cows

**DOI:** 10.3389/fvets.2025.1444674

**Published:** 2025-02-26

**Authors:** Osamu Ichii, Teppei Nakamura, Masaya Hiraishi, Takashi Namba, Md. Zahir Uddin Rubel, Takuya Umeyama, Megumi Asai

**Affiliations:** ^1^Laboratory of Anatomy, Department of Basic Veterinary Sciences, Faculty of Veterinary Medicine, Hokkaido University, Sapporo, Japan; ^2^One Health Research Center, Hokkaido University, Sapporo, Japan; ^3^Laboratory of Laboratory Animal Science and Medicine, Department of Applied Veterinary Sciences, Faculty of Veterinary Medicine, Hokkaido University, Sapporo, Japan; ^4^Business Innovation Department, Furukawa Sangyo Kaisha, Ltd., Tokyo, Japan

**Keywords:** chlorous acid water, disinfection, dairy cows, surgical site infection, veterinary medicine, skin, paralumbar fossa, farm animals

## Abstract

Disinfection is crucial for preventing surgical site infections. Recently, the effectiveness of sanitizers using chlorous acid (HClO_2_) under conditions rich in organic matter has been reported, and chlorous acid water (CAW) has been approved as a food additive. This study evaluated the potential of CAW as a new presurgical disinfectant for cattle. The experiments were performed on the paralumbar fossa of cattle in Sapporo during March (winter to spring) and August (summer). Colony-forming units (CFUs) of standard plate count bacteria (SPCB), *Enterococcus faecalis* (EF), *Pseudomonas aeruginosa*, *Escherichia coli*, and *Staphylococcus* spp. (SP) were analyzed as indicators of bacterial load. SPCB and SP were abundantly detected, exceeding 6 log_10_ CFU/100 g on clipped hair and 6 log_10_ CFU/100 cm^2^ on the skin immediately after clipping, with no significant seasonal differences. The bacterial load on the skin was evaluated at three time points: after clipping, cleansing, and disinfection. Clipping and cleansing with liquid soap were common procedures, following this, either the standard disinfection protocol using 7.5% iodine scrub for 1 min, 10% povidone-iodine for 5 min, and 70% alcohol for 5 min (SPA), or a modified protocol using CAW with contact times of 15, 10, or 5 min (CAW15, CAW10, CAW5) were performed separately. The cleansing procedure significantly reduced the SPCB, EF, and SP on the skin after clipping, and all disinfection methods significantly decreased the SP after cleansing. Draping significantly enhanced the disinfection efficiency of the SPA, CAW10, and CAW5 protocols. The CAW procedure did not alter skin histology in the paralumbar fossa or udder compared to 10% povidone-iodine or 70% alcohol. Our data suggest that the disinfection method using CAW is useful and comparable to routine disinfection methods and might reduce the time required for presurgical disinfection in farm fields.

## 1 Introduction

Disinfection of the surgical site is crucial for reducing bacterial contamination that can result in surgical site infection (SSI). In both human and veterinary medicine, povidone-iodine (PVP-I), chlorhexidine gluconate, alcohol, and their combinations are commonly used for general skin disinfection before surgical procedures (1, 2). PVP-I, chlorhexidine gluconate, and alcohol exhibit bactericidal effects through the oxidizing action of iodine ions, bacteriolytic action, and protein-coagulating action, respectively. In human cases, the day before or the day of surgery, the patients take a bath or shower, or wipe the skin to remove dirt and ensure adequate cleansing. Surgical clippers are occasionally used to remove hair, but shaving is currently avoided because of the risk of skin damage leading to SSI, and shaving or short clippers are not recommended in animals for the same reasons (3–5). Finally, in humans, the surgical site is wiped with a cotton ball filled with a disinfectant. To prevent SSI, these procedures are performed in the operating or disinfection room where microbiological cleanliness is maintained.

In veterinary medicine at present, the disinfection control performed is equivalent to that performed in human medicine, especially for surgeries. In particular, well-equipped operating or disinfection rooms are maintained in veterinary hospitals for dogs, cats, and racehorses. Unlike humans, almost all animals have abundant hair coats carrying abundant bacteria, and the hair of farm or wild animals are dirty with soil or excrement (6 log_10_ colony-forming units [CFU]/cm^2^ in cow-clipped hairs) (6); therefore, it is important for surgical treatment to clean and disinfect the skin after clipping (5). In veterinary medicine, a human hand disinfection method based on the traditional brushing methods by Fürbringer, Grossich, or their modified procedures, has been used for disinfection of the surgical site (7). Although the disinfectant and the time of exposure differ among animals, veterinarians, and hospitals, the surgical site is cleansed with a surfactant, flushed with water, washed with a surgical scrub containing PVP-I or chlorhexidine, and then sprayed with PVP-I and/or an alcohol-based reagent (8–10).

Farm animal surgery is performed in standing or dorsal recumbency in the operating room (11, 12). In addition, several cases are performed using treatment stalls in conventional rearing spaces in the field. In cattle, the paralumbar fossa is a common site for abdominal surgery and is surgically incised for cesarean section, abomasal displacement, and other gastrointestinal or urogenital diseases (11, 12). The paralumbar fossa is disinfected using a brushing method based on Fürbringer’s or Grossich’s procedures, similar to other animals (8). Brushing methods require time and staff; therefore, it is important to consider a quicker and easier disinfection method with high disinfection efficiency to reduce the contamination risk of falling bacteria and the burden on animals as well as veterinary staff. Several veterinarians have tried to reduce the operating time by changing the exposure time to disinfectants (8, 9, 13); Bourel et al. reported a disinfection method comprising two 90-s periods of cleansing and scrubbing, with 3 passages of 0.5% chlorhexidine gluconate in 70% in isopropyl alcohol solution (approximately, total 4 min) (8).

The present study focused on the usefulness of chlorous acid, HClO_2_, for disinfecting animal skin, because HClO_2_-based sanitizers have been reported to be more stable than NaClO under organic-matter-rich conditions. They contain chlorinated oxides such as HClO_2_ or dissolved chlorine dioxide (ClO_2_) which exhibit microbicidal activity. Recently, chlorous acid-based sanitizers have been used in food and environmental sanitation, and other studies have reported their microbicidal effects on a wide range of microorganisms, including yeast and spore-forming bacteria, such as *Escherichia coli* (EC), *Staphylococcus aureus*, *Campylobacter jejuni*, *Clostridium difficile* spores, *Candida albicans*, spore-forming *Bacillus*, and *Paenibacillus* species, as well as human norovirus and feline calicivirus (14–16). Chlorous acid water (CAW) was approved as a food additive by the Ministry of Health, Labor, and Welfare of Japan in 2013, and HClO_2_-based disinfection was classified as a second-class OTC drug in 2019. Thus, HClO_2_-based sanitizers, especially CAW, would have the potential to be effective disinfection reagents; however, the effectiveness of animal skin disinfectants is unclear, especially for future applications in clinical procedures in veterinary medicine.

Therefore, the present study evaluated the potential of CAW as a presurgical disinfectant in cattle. Our data suggest that disinfection using CAW is useful and comparable to routine disinfection methods, and might lead to a reduction in the time required for presurgical disinfection in farm fields.

## 2 Materials and methods

### 2.1 Animals and environment

Female Holstein cows were maintained at the experimental farm of the Field Science Center for Northern Biosphere, Hokkaido University. All animal experiments were approved by the Institutional Animal Care and Use Committee of Hokkaido University (approval no. 22-0110, 3/17/2023). The experiments were performed in March 2023 (temperature 11.5 ± 0.7°C, humidity 54.4 ± 2.2%) and August 2023 (temperature 27.5 ± 0.5°C, humidity 74.1 ± 0.5%) at cattle housing, considering seasonal effects. Monthly changes in temperature and humidity were based on information published by the Japan Meteorological Agency (https://www.jma.go.jp/jma/indexe.html; Tokyo, Japan).

### 2.2 Routine disinfection procedure based on Fürbringer’s or Grossich’s method

**Figure 1:**
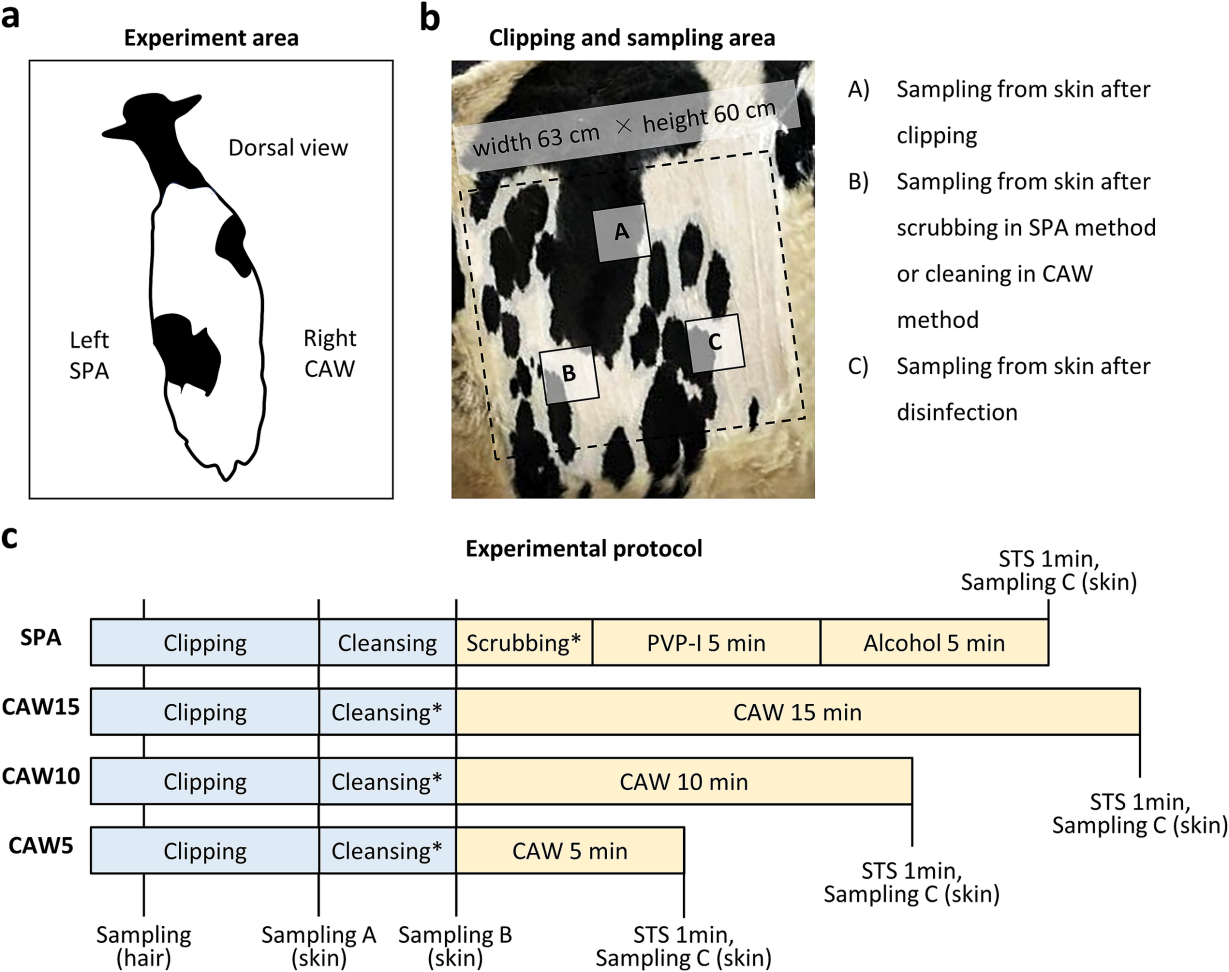
Experimental protocol in this study. **(a)** Dorsal view of the cow in the present experiment. Left or right paralumbar fossae are used for standard disinfection protocol using scrubbing, povidone iodine, and alcohol (SPA) or modified protocol using chlorous acid water (CAW), respectively. **(b)** Clipped area for bacteria sampling. Areas (A), (B), and (C) are used for sampling from skin after clipping, scrubbing using the SPA method or cleaning using the CAW method, and disinfection, respectively. **(c)** Experimental protocol. Clipping and cleansing using liquid soap is commonly performed in each protocol. After washing with tap water in cleansing or scrubbing, the experimental area is wiped with sterile gauze. Sampling A–C is corresponding to area A–C in panel **(b)**. After disinfection, 0.1% mol/L sodium thiosulfate (STS) is sprayed on each area. *: Draping is performed in the experiment show in Figure 4. PVP-I, povidone iodine.

Figure 1 summarizes the experimental procedure, including routine disinfection using scrubbing, povidone-iodine, and alcohol (SPA). All experimental procedures were conducted within the cattle housing. The cows were randomly assigned to the trial without specifically considering their lactation status, encompassing both dry and lactating periods. The experiments were performed during specific time frames: in March from 10:00 a.m. to 4:00 p.m., and in August from 9:00 a.m. to 4:00 p.m. The cows were tied to a stall and their tails were secured with a string to prevent movement. The left paralumbar fossa (width, 63 cm; height, 60 cm) was clipped using clippers (Xperience, Heiniger; Herzogenbuchsee, Switzerland) equipped with blade no. 53a-23 (1 mm after clipping; Heiniger). Then, the skin surface of the paralumbar fossa was cleaned with 20 mL of liquid soap using a disposable polypropylene brush (7.5 × 10 × 5 cm) and a small amount of tap water for 1 min. The soap bubbles were rinsed with tap water, and the clipped area was wiped with sterilized gauze (30 × 30 cm, Iwatsuki; Tokyo, Japan) to prevent contamination from the unclipped area. The clipped area was washed with a new polypropylene brush using 7.5% iodine scrub solution (88.5 g, Shionogi; Osaka, Japan) for 1 min. The scrubs were rinsed with tap water and the clipped area was wiped with sterilized gauze (Iwatsuki). In some cases, the clipped area was covered with a sterile surgical drape (90 × 90 cm; Nissho Sangyo; Tokyo, Japan). Then, 10% PVP-I (55 mL, Fujita Pharmaceutical Co., Ltd.; Tokyo, Japan) was sprayed and contacted for 5 min; then, 70% alcohol (55 mL, Japan Alcohol Corporation; Tokyo, Japan) was sprayed and contacted for 5 min. To neutralize the disinfectants, 0.1 mol/L sodium thiosulfate (STS) was sprayed and contacted for 1 min. Clipping and cleansing were performed by 2 experimenters, whereas sampling and disinfectant spraying were performed by 1–2 other experimenters.

### 2.3 Modified disinfection procedure using CAW

As summarized in Figure 1, the procedure was the same as that in the routine SPA method, but we sprayed 1,000–8,000 ppm (free available chlorine = 25–200 mg/L) of Klorus disinfectant water (55 mL, PURGATIO Inc., Tokyo, Japan) after washing with liquid soap, rinsing with tap water, and wiping with sterilized gauze on the right paralumbar fossa of the same cow used in the SPA method. The concentration used was determined through a preliminary experiment (Supplementary Figure S1). The contact times of the CAW were examined at 5, 10, and 15 min. In some cases, the clipped area was covered with a sterilized surgical drape (90 cm × 90 cm; Nissho Sangyo) after spraying with CAW. To neutralize the disinfectants, 0.1 mol/L STS was sprayed and contacted for 1 min. Clipping and cleansing were performed by 2 experimenters, whereas sampling and disinfectant spraying were performed by 1–2 other experimenters.

### 2.4 Sample collections

Hair or bacteria were collected from the skin surface layers using rayon cotton swabs (Wipe Check II; Eiken Chemical; Tokyo, Japan). The swab area was measured using a sterile frame (10 × 10 cm; AS ONE Corporation; Osaka, Japan). As shown in Figure 1, sample collections of swabbing were performed for 3 times; (A) after clipping, (B) after wiping with gauze after liquid soap cleansing, and (C) after spaying the STS for both the SPA and CAW methods. Samples were collected from different areas (A), (B), and (C).

### 2.5 Microbiological examination

All samples were appropriately diluted for saline, and 0.1 mL of diluted samples were used.

#### 2.5.1 Standard plate count bacteria

Diluted sample was spread on standard method agar (Nissui Pharmaceutical Co. Ltd.; Tokyo, Japan) using sterilized glass beads and incubated at 35°C for 24–48 h to enumerate the surviving bacteria.

#### 2.5.2 Enterococcus faecalis

Diluted sample was spread on EF agar base (Nissui Pharmaceutical Co. Ltd.) with 0.0015% 2,3,5,-triphenyltrtrazolium chloride using sterilized glass beads and incubated at 35°C for 24–48 h. Colonies with colors ranging from pink to dark brown were enumerated as surviving EF.

#### 2.5.3 Pseudomonas aeruginosa

Diluted sample was spread on NAC agar (Eiken Co. Ltd.; Tokyo, Japan) using sterilized glass beads, and incubated at 35°C for 24–48 h; yellow-greenish fluorescent colonies were enumerated as the surviving *PA*.

#### 2.5.4 EC

Diluted sample was spread on X-MG agar medium (Shimadzu Diagnostics Corporation; Tokyo, Japan) with sterilized glass beads and incubated at 35°C for 18–22 h; blue colonies were enumerated as the surviving *EC*.

#### 2.5.5 *Staphylococcus* spp.

Diluted sample of the diluted sample was spread on mannitol salt agar (Nissui Pharmaceutical Co. Ltd.) with added egg yolk using sterilized glass beads and incubated at 35°C for 24–48 h; colonies on the medium were enumerated as the surviving *Staphylococcus* spp.

### 2.6 Evaluation of environmental factors

**Figure 2:**
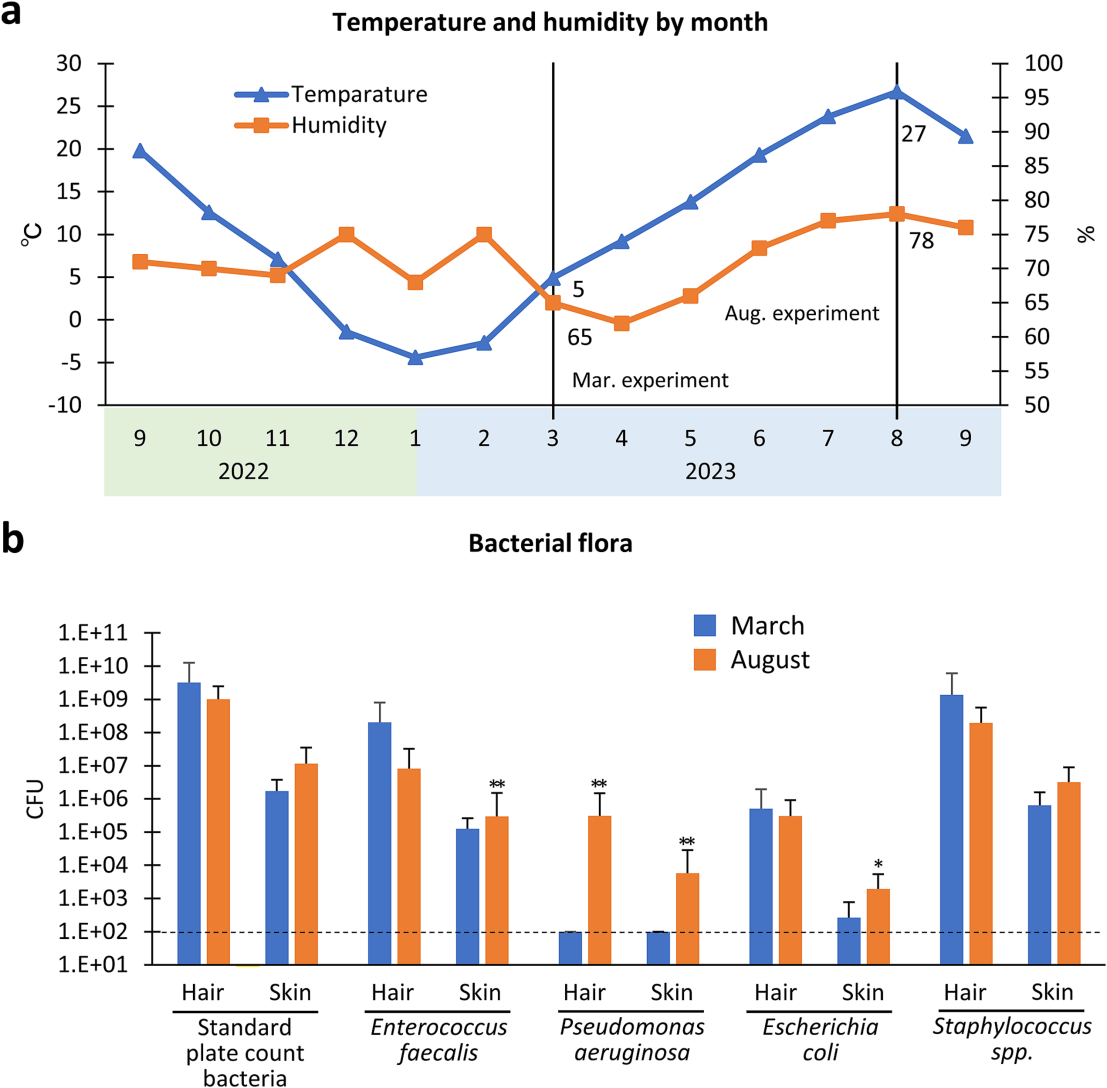
Seasonal differences of bacterial flora in the hair and skin of cows. **(a)** Changes in temperature and humidity according to month (from September 2022 to September 2023) in Sapporo city, Japan. The experiments are conducted in March 2023 and August 2023. Values = mean. **(b)** Bacterial flora of hair and skin in March or August 2023. *, **: Indicates significances with respect to March in hair or skin, as determined using the Mann–Whitney *U* test (*p* < 0.05, 0.01, respectively, Values = mean ± standard deviation). CFU, colony forming unit/100 g hair or/100 cm^2^ skin. Dotted line indicate the detection limit (DL). *n* = 30 samples from 15 cows in March, *n* = 48 samples from 23 cows in August.

The experimental site, Sapporo, experiences significant seasonal variations in temperature and humidity levels between summer and winter (Figure 2). Generally, bacteria thrive in the warmer summer conditions. Consequently, this study evaluated these environmental factors and their impact on bacterial growth. To assess the potential bacterial contamination of the experimental procedure due to environmental factors, we examined SPBC or coliform bacteria. To evaluate airborne bacterial contamination, standard method agar plates were placed at four different locations within the farm, and this process was repeated four times. The plates were exposed with their lids open for 5 min, and the resulting bacterial colonies were counted after incubating the plates at 35°C for 24–48 h. For the tap water analysis, the samples were appropriately diluted using saline solution, and 1 mL of each dilution was transferred to a sterile plastic petri dish. Subsequently, 20–25 mL of sterilized medium, either deoxycholate agar (Nissui Pharmaceutical Co. Ltd.) for coliform bacteria or standard method agar (Nissui Pharmaceutical Co. Ltd.) for SPCB, was poured into the dishes. The mixtures were gently agitated, allowed to solidify, and then incubated at 35°C for 18–48 h to count the proliferating microorganisms.

### 2.7 Histological analysis of skin exposed to disinfectant

For histological analysis, one adult cow that was euthanized in a separate experiment was used, and the skin of the paralumbar fossa was incised within minutes after euthanasia. Alcohol (70%), PVP-I, or CAW was sprayed onto the skin surface or incised area and contacted for 15 min. The skin was collected and fixed in a mixture of formalin, acetic acid, and absolute ethanol (volume ratio, 10:5:85) for 24 h at room temperature. After dehydration using alcohol, tissues were embedded in paraffin and cut into sections (4 μm thick), including the region exposed to disinfectants. The deparaffinized sections were stained with hematoxylin–eosin (H&E).

### 2.8 Statistical analysis

Results were expressed as the mean with or without standard deviation and analyzed statistically with nonparametric methods using IBM SPSS Statistics 28.0.1.0 (142) (IBM; Armonk, NY, United States). For bacterial analysis, colony-forming units (CFU) were expressed as CFU/100 g in the hair and CFU/100 cm^2^ in the skin. The reduction ratio of CFU in the skin samples after cleansing or disinfection to those immediately after clipping was also calculated. The Mann–Whitney *U* test was used to compare the two populations (*p* < 0.05). The Kruskal-Wallis test was used to compare the three populations, and multiple comparisons were performed using Scheffé’s method when a significant difference was observed (*p* < 0.05).

## 3 Results

### 3.1 Overview of experimental protocol and time required for each procedure

Figure 1 illustrates the experimental procedures used in this study. The left and right paralumbar fossae were used for SPA and CAW disinfection, respectively (Figure 1a). Both experimental areas were clipped, and skin swab sampling for bacterial analysis was performed at different timing according to each purpose as shown in Figure 1b; sampling from skin after clipping (A), cleansing (B), or reaction termination of disinfects with STS (C). Figure 1c shows the experimental procedure from clipping to disinfection and reaction termination by STS for each time course. Clipping and cleansing were common in all procedures; however, the CAW method could skip scrubbing. Furthermore, CAW10 and CAW5 (contact times of 10 and 5 min, respectively) were shorter than those of the SPA method, which required scrubbing and exposure to PVI-I and alcohol (5 min each). CAW15 (contact time of 15 min) was the longest procedure in this study.

### 3.2 Seasonal differences of bacterial flora in the clipped hair and skin of cows

The present experiments were performed in March (winter to spring) and August (summer), 2023 in Sapporo city, Japan. Figure 2a shows the monthly changes in temperature and humidity in Sapporo based on data published by the Japan Meteorological Agency. Actual temperature and humidity of experimental farm in Hokkaido University was 11.5 ± 0.7°C, 54.4 ± 2.2% on March and temperature 27.5 ± 0.5°C, 74.1 ± 0.5% on August during this experiment.

Figure 2b shows the CFU differences in bacterial flora cultured from clipped hair and skin between the March and August experiments (note the difference in value calculation, CFU 100 g hair and/or 100 cm^2^ skin). Hair collected in August showed significantly higher CFU in PA than hair collected in March (*p* < 0.01). Furthermore, the CFU in skin samples were significantly higher in August than in March for EF (*p* < 0.01), PA (*p* < 0.01), and EC (*p* < 0.05). Thus, these data indicate that EF, PA, and EC on the skin surface increased in summer, but SPCB and *Staphylococcus* spp. on the skin, which showed higher CFU (over 6 log_10_ CFU/cm^2^) than other spp., were comparable between the two seasons.

### 3.3 Effect of disinfection protocols on skin bacterial flora

The effective concentration of CAW was examined in a preliminary study (Supplementary Figure S1). CFU or reduction ratio of CFU in skin samples after cleansing or disinfection to those immediately after clipping (reduction % vs. skin) were compared between SPA and modified protocol using CAW100% (8,000 ppm), CAW50% (4,000 ppm), CAW25% (2,000 ppm), and CAW12.5% (1,000 ppm). For SPCB, CAW 100% showed comparable disinfection ability to SPA. In the condition blow 50% of CAW, CFU of SPCB tended to be higher that of SPA. For *Staphylococcus* spp., in the conditions below 25% of CAW, CFU tended to be higher compared to SPA. Based on these results, we proceeded with the experiment using 100% to guarantee the highest disinfection performance of CAW.

**Figure 3:**
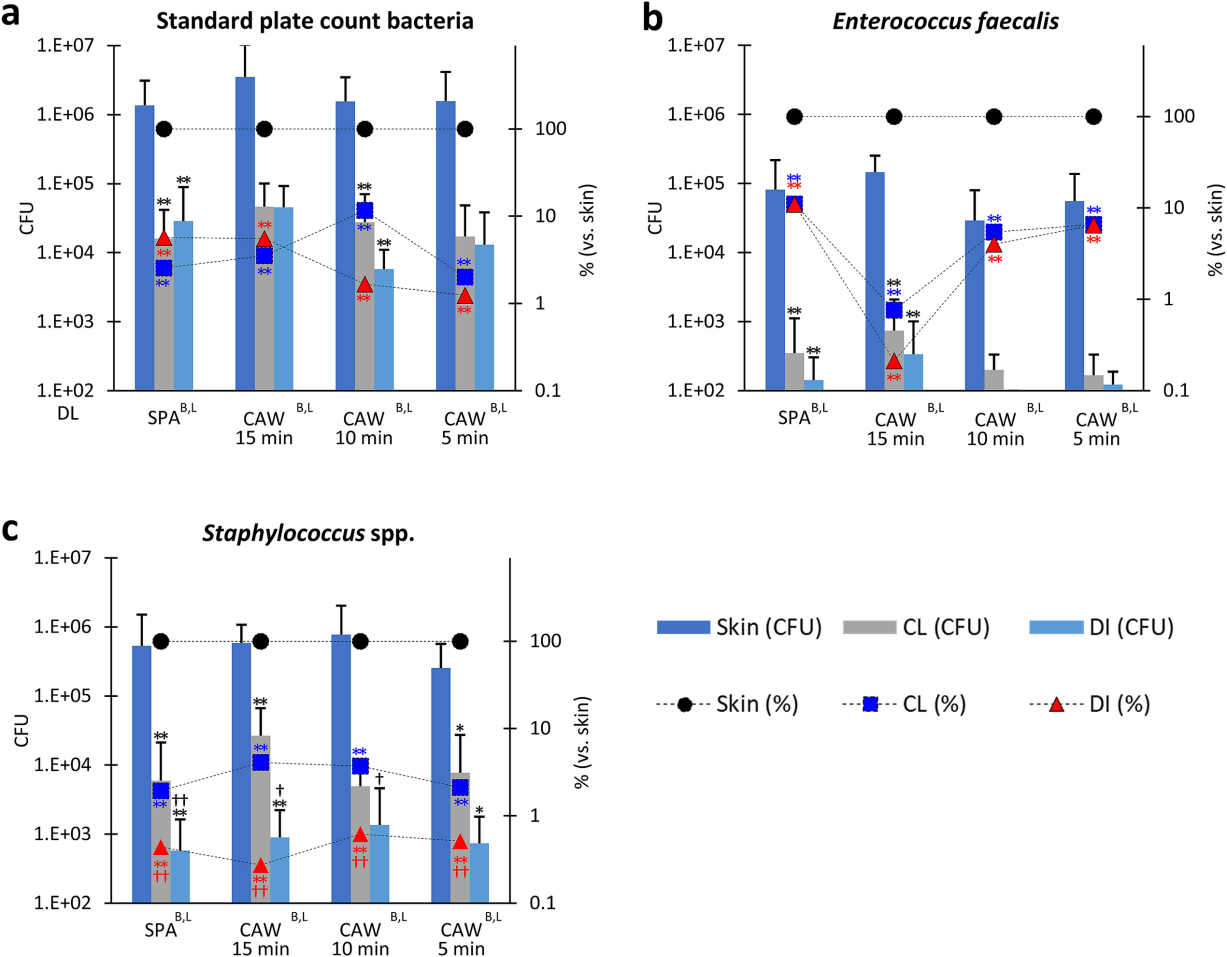
Bacterial flora in the skin of cows. **(a)** Standard plate count bacteria. **(b)** *Enterococcus faecalis*. **(c)** *Staphylococcus* spp. Standard disinfection protocols using scrubbing, povidone iodine, and alcohol (SPA), and a modified protocol using chlorous acid water for 15, 10, and 5 min (CAW15, 10, and 5) were compared. Bar graphs represent colony-forming units (CFU). The line graph shows the ratio of skin samples after clipping to those after cleaning (CL) and disinfection (DI). B, L: Significance determined using the Kruskal-Wallis test (*p* < 0.01) in bar and line graphs, respectively. *, ** (black): Indicates significances with respect to skin CFU, as determined using Scheffé’s method (*p* < 0.05, 0.01, respectively). ** (blue): Indicates significances with respect to skin CL (%), as determined using Scheffé’s method (*p* < 0.01, respectively). ** (red): Indicates significances with respect to skin DI (%), as determined using Scheffé’s method (*p* < 0.01, respectively).^†, ††^ (black): Indicates significance with CL (CFU), as determined using the Mann–Whitney *U* test (*p* < 0.05, 0.01, respectively). ^†, ††^ (red): Indicates significance with CL (%), as determined using the Mann–Whitney *U* test (*p* < 0.05, 0.01, respectively). Bar graph: values = mean ± standard deviation. Line graph: values = mean. The dotted line indicates the detection limit (DL). *n* = 28 samples from 20 cows in SPA, *n* = 8 samples from 7 cows in CAW15, *n* = 9 samples from 8 cows in CAW10, *n* = 9 samples from 6 cows in CAW5.

As shown in Supplementary Figure S2, the CFUs of PA and EC were lower, and the differences in each disinfection method were not clear; therefore, we focused on other bacteria, as shown in Figure 3. The disinfection efficiencies of SPA, CAW15, CAW10, and CAW5 were compared. CFU or reduction % vs. skin was also evaluated for each examined bacterial species using the combined data obtained in March and August. For SPCB, all methods reduced the CFU of skin samples after cleansing or disinfection, and statistically significant reductions were observed for SPA and CAW10 (*p* < 0.01). For the reduction % vs. skin in SPCB, significant differences were observed in skin samples after cleansing or disinfection for all methods (*p* < 0.01). Furthermore, no significant differences between cleansing and disinfection were observed for any of the methods. For EF, all methods reduced the CFU of skin samples after cleansing or disinfection, and statistically significant reductions were observed for SPA and CAW15 (*p* < 0.01). Furthermore, disinfection tended to reduce CFU compared to cleansing. For the reduction % vs. skin, significant differences were observed in samples after cleansing or disinfection for all methods (*p* < 0.01), and significant differences between cleansing and disinfection were observed for SPA (*p* < 0.05). These data indicate the importance of cleansing to reduce SPCB on the cow skin surface.

For *Staphylococcus* spp., all methods significantly reduced the CFU of the skin or the ratio to skin samples after cleansing or disinfection, with statistical significance for SPA, CAW15 (*p* < 0.01), and CAW5 (*p* < 0.05). Furthermore, reduction % vs. skin also significantly decreased after cleansing or disinfection in all methods (*p* < 0.01). In particular, disinfection reduced both CFU and reduction % compared to cleansing, and statistical significance was detected in the CFU of SPA (*p* < 0.01), CAW15, and CAW10 (*p* < 0.05), and in the reduction % in all methods (*p* < 0.01). These data indicate that disinfection using either SPA or CAW was effective in reducing *Staphylococcus* spp. after cleansing.

### 3.4 Effect of draping to disinfection efficiency in the farm field

**Figure 4:**
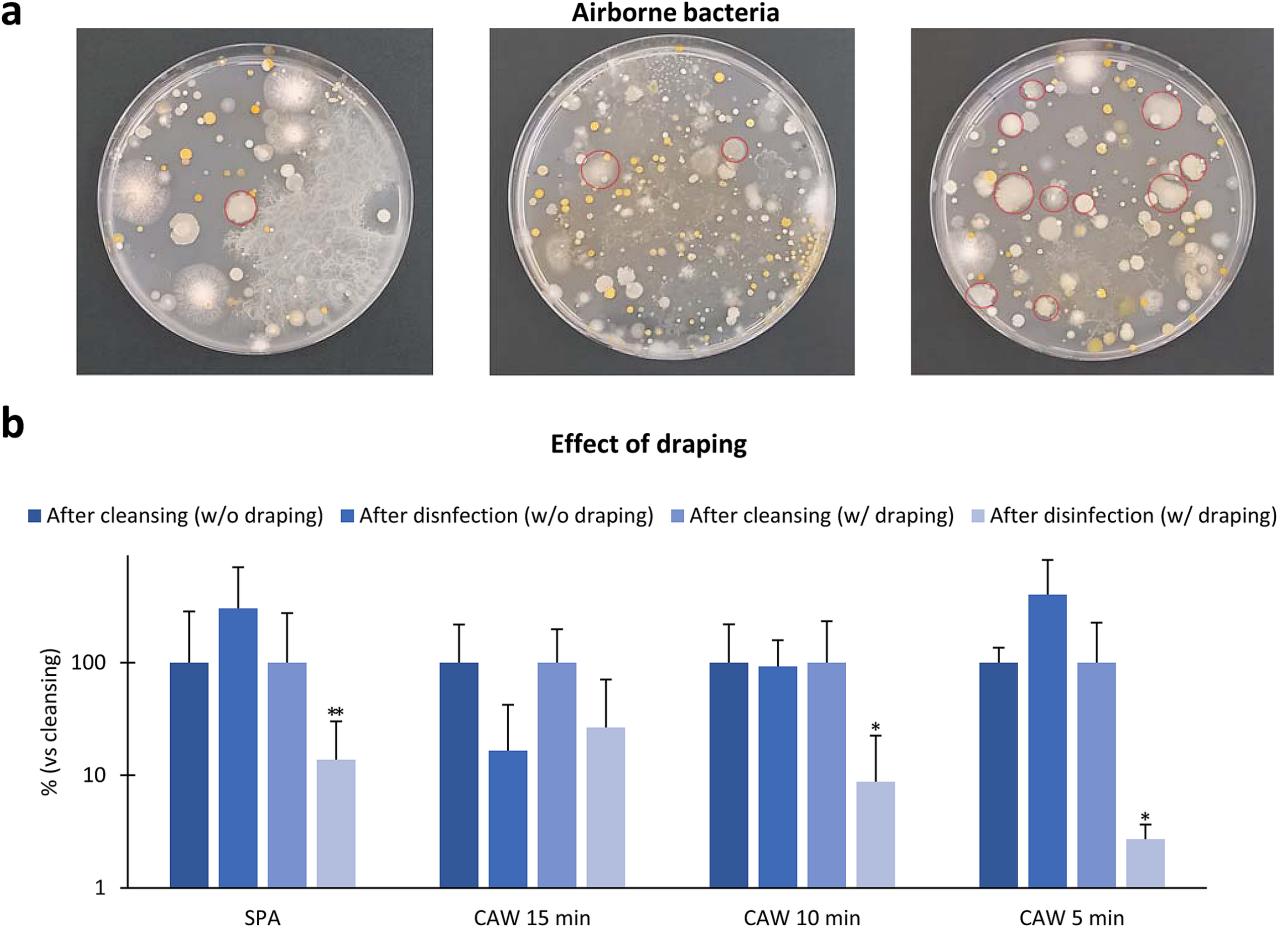
Effect of environmental factors. **(a)** Bacteria samples collected from three different area in farm fields. The plates are exposed with their lids open for 5 min, and the resulting bacterial colonies are counted after incubating the plates at 35°C for 24–48 h. Numerous colonies are observed, and red circles indicate *Bacillus* spp. colonies. **(b)** Effect of draping to skin bacterial flora. Data represents the ratio change of standard plate count bacteria in skin sample after disinfection without or with draping to those after cleansing. *, **: Indicates significances with respect to skin DI (%), as determined using the Mann–Whitney *U* test (*p* < 0.05, 0.01, respectively). Values = mean ± standard deviation. *n* = 12 samples from 10 cows in SPA, *n* = 4 samples from 4 cows each in CAW15 and CAW10, *n* = 4 samples from 3 cows in CAW5, without draping condition. *n* = 12 samples from 12 cows in SPA, *n* = 4 samples from 4 cows each in CAW15, CAW10, and CAW5, with draping condition.

As shown in Figure 4a, numerous colonies, including *Bacillus* spp., derived from airborne bacteria were observed in the samples collected from the different area (265 ± 213 colonies, *n* = 11, 5 min exposure, 24–48 h). As the experiments shown in Figure 3 were performed without draping, we verified the effect of draping on the skin bacterial flora during the disinfection protocol (Figure 4b). Draping with the disinfection protocol using SPA, CAW15, CAW10, and CAW5 decreased the ratio of CFU in skin samples immediately after clipping, and significant differences were observed in SPA (*p* < 0.01), CAW10, and CAW5 (*p* < 0.05), emphasizing the importance of draping during the disinfection procedure.

### 3.5 Histology of skin after experimental procedures

**Figure 5:**
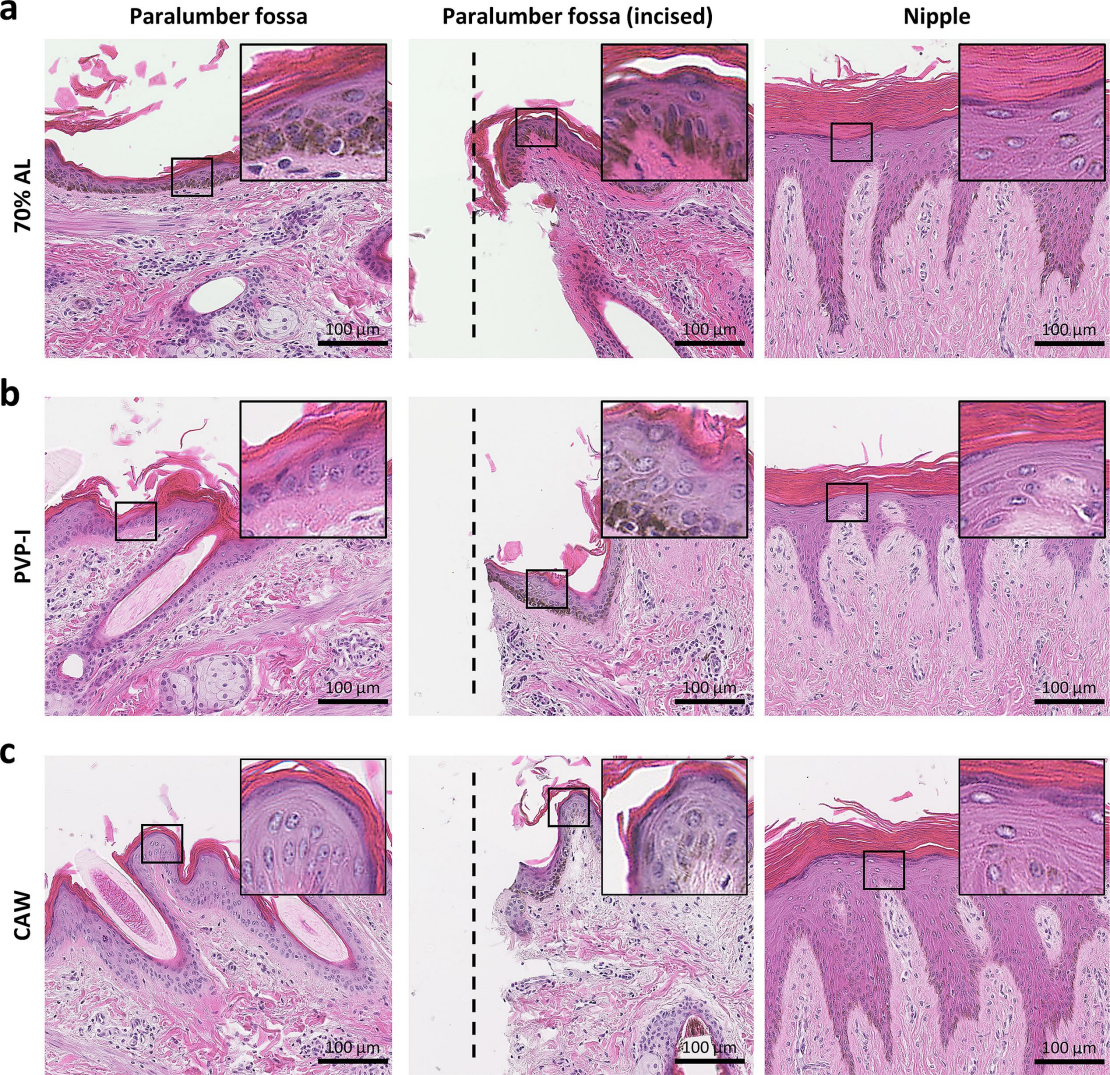
Histology of skin after experimental procedures. **(a)** Histology of the skin area sprayed with 70% alcohol (AL). **(b)** Histology of the skin area sprayed with povidone iodine (PVP-I). **(c)** Histology of the skin area sprayed with chlorous acid water (CAW). Samples were collected from each area 15 min after spraying. In the incised area of the paralumbar fossa, each disinfectant was sprayed directly onto the incision wound using a scalpel. On histological examination, skin structures, especially those of the epidermis, such as the cornified stratified squamous epithelium, were well-preserved without remarkable structural changes. The insets magnify the squared areas. Hematoxylin and eosin staining.

In Figure 5, we examined the histological features of skin in the paralumbar fossa or udder after exposure to 70% alcohol, PVP-I, or CAW for 15 min; these procedures were performed without scrubbing. As shown in the panels, skin histological structures, including the cornified stratified squamous epithelium and dermis, did not change after any of the procedures (Figures 5a–c). We also examined the histology of the incised skin after direct exposure to each reagent, but no clear histological changes due to reagent contact were observed in the incised area. Furthermore, we applied these methods to udders, assuming the application of CAW for disinfection during milking. The epidermis of the udder was thicker and more highly cornified than that of the paralumbar fossae, and no histological changes were observed upon exposure to each reagent.

## 4 Discussion

In this study, we evaluated the potential of CAW as a pre-surgical disinfectant for cattle skin. Our data suggest that disinfection using CAW is useful and comparable to routine veterinary methods that use a combination of scrubbing, PVP-I, and alcohol. Importantly, our disinfection method using CAW could lead to a reduction in the operation time required for presurgical disinfection in farm fields, as it eliminates the need for scrubbing. Furthermore, our data emphasize the importance of clipping, cleansing, and draping for effective disinfection of animal skin.

First, we examined seasonal changes in the bacterial flora in the hair and skin of cattle. In general, hair contains numerous bacteria as reported previously (6). Several studies have indicated that animal hair provides a favorable environment for bacteria growth (17). High variability in bacteria was also observed between different skin regions within the same dog, with a higher number of bacterial species observed on haired skin than on poorly haired skin or mucocutaneous junctions (18). The examined bacterial species, including SPCB, EF, and *Staphylococcus* spp. (over 6 log_10_ CFU/g), were more abundant than PA and EC (under 6 log_10_ CFU/g) in clipped cattle hairs. These data also strongly suggest that hair clipping is a crucial first step in skin disinfection in cattle, although shaving or short clippers are not recommended for animals because of the risk of skin damage leading to SSI (3–5). Cattle skin also contained abundant SPCB and *Staphylococcus* spp. (over 6 log_10_ CFU/100 cm^2^) compared to others (under 6 log_10_ CFU/100 cm^2^), and this tendency was similar to that of hair bacterial flora. However, the CFU of EF, PA, and EC were significantly higher in August than in March, and PA in hair showed significantly higher CFU than in March. Bacterial populations on the human skin are significantly affected by high-temperature and high-humidity environments compared to moderate-temperature and low-humidity environments (19). These data emphasize the importance of hair clipping and skin cleansing and disinfection according to environmental changes, especially under high temperature or humidity conditions.

Among all examined procedures, cleansing with liquid soap and a polypropylene brush, followed by rinsing with tap water, significantly decreased the CFU on cattle skin. In fact, the CFU counts of the examined bacteria, except for *Staphylococcus* spp., were comparable between the samples after cleansing and disinfection, regardless of whether SPA or CAW was used. Regarding the concentration of CAW, we determined that 8,000 ppm (free available chlorine = 200 mg/L) would be stable for the quality of presurgical disinfectant because 4,000 ppm (free available chlorine = 100 mg/L) decreased the disinfection efficiency of SPCB compared to 8,000 ppm in a preliminary study (see Supplementary Figure S1). Regarding the exposure time of the CAW, there was no remarkable difference among 5, 10, and 15 min of exposure for the examined bacterial CFU, and their disinfection efficiency was comparable with that of the SPA. Furthermore, disinfection using CAW can eliminate the scrubbing procedure required for SPA. The disinfection efficiency of CAW can be realized based on the chemical properties of HClO_2_ in CAW, which can act under organic-matter-rich conditions (14–16). Therefore, the use of CAW (8,000 ppm) for 5 min after hair clipping and liquid soap cleansing can shorten the operation time for pre-surgical disinfection.

In the present study, the CFU of *Staphylococcus* spp. were significantly decreased in cattle skin disinfected with SPA and CAW compared to those cleansed with liquid soap. *Staphylococcus* spp. currently comprises 81 species and subspecies, with most members of the genus being mammalian commensals or opportunistic pathogens that colonize niches, including the skin (20). Several *Staphylococcus* spp. can cause serious pathological problems in human and veterinary medicine. *Staphylococcus epidermidis*, a normal component of the epidermal microbiota, can lead to biofilm contamination of medical devices (21). Especially, in dairy cows, *S. aureus* is a major cause of mastitis, resulting in significant economic losses. Importantly, *S. aureus* infections are major risk factors for SSI in animals (20); therefore, CAW disinfection before surgery can contribute to reducing the risk of SSI, similar to the routine SPA method.

In the present study, we demonstrated the effectiveness of draping the surgical area (22), which can protect against bacterial contamination. In veterinary medicine, draping the surgical area as soon as possible after cleansing and disinfection is crucial in practical on-site disinfection scenarios such as on farms. Importantly, the CAW method, with 5 min of exposure, can reduce the chance of contamination from falling bacteria because it can skip the scrubbing time. In fact, a 15 min exposure to CAW did not result in a more effective disinfection efficiency compared to 5 min. This could be explained by the increased chance of contamination from the environment, such as falling bacteria or dripping water from hair surrounding the surgical area. Therefore, the CAW disinfection method can increase its disinfection efficiency by immediately draping and wiping off the surrounding area using a sterile gauze.

## 5 Limitations

CAW has been applied in food and environmental sanitation, and the microbicidal effects on a wide range of microorganisms, including yeast, EC, *S. aureus, C. jejuni, C. difficile* spores, *C. albicans*, spore-forming *Bacillus*, and *Paenibacillus* species, as well as human norovirus and feline calicivirus have been reported (14–16). Because cattle can contract dermatophytosis, it is important to evaluate their susceptibility to fungi. Furthermore, the present study demonstrated the usefulness of CAW for skin disinfection. No histological changes were observed in the skin of the paralumbar fossa and udders of cattle after exposure to CAW for 15 min. However, the residual time in tissues has not yet been evaluated because the reaction was stopped with STS to guarantee an accurate reaction time. Furthermore, stopping the experimental reaction with STS cannot be used to evaluate the sustained effects of the CAW, which might cause an underestimation of its disinfection efficiency. For example, a recent study showed that 1 h after application, the bacterial reduction was better sustained with chlorhexidine than with ethanol, but no difference was found between chlorhexidine and isopropyl alcohol (23). For further applications of CAW in veterinary medicine, such as multiple spraying in the surgical area, cattle teat disinfection, or fogging of farm areas, the residual time in each region should be accurately evaluated in future studies. Effects on skin when used with electrocautery should also be evaluated (24).

In conclusion, the present study suggests that disinfection using CAW is useful and comparable to routine SPA disinfection methods and might lead to a reduction in the operation time required for presurgical disinfection in farm fields.

## Data Availability

The original contributions presented in the study are included in the article/Supplementary material, further inquiries can be directed to the corresponding author.

## References

[1] Darouiche RO Wall MJ Itani KMF Otterson MF Webb AL Carrick MM. Chlorhexidine–alcohol versus povidone–iodine for surgical-site antisepsis. N Engl J Med. (2010) 362:18–26. doi: 10.1056/NEJMoa0810988, PMID: 20054046

[2] Maxwell EA Bennett RA Mitchell MA. Efficacy of application of an alcohol-based antiseptic hand rub or a 2% chlorhexidine gluconate scrub for immediate reduction of the bacterial population on the skin of dogs. Am J Vet Res. (2018) 79:1001–7. doi: 10.2460/ajvr.79.9.1001 30153054

[3] Messiaen Y Maclellan JD Pelsue DH. Evaluation of the number of colony forming units on the skin of dogs after clipping the hair with two sizes of clipper blades. Am J Vet Res. (2019) 80:862–7. doi: 10.2460/ajvr.80.9.862, PMID: 31449448

[4] Lane C. Preventing surgical site infections: equine surgical site preparation. Vet Nurse. (2016) 7:151–5. doi: 10.12968/vetn.2016.7.3.151

[5] Bédard S Desrochers A Fecteau G Higgins R. Comparison of four protocols for preoperative preparation in cattle. Can Vet J. (2001) 42:199–203. PMID: 11265188 PMC1476472

[6] Reid CA Avery SM Hutchison ML Buncic S. Evaluation of sampling methods to assess the microbiological status of cattle hides. Food Control. (2002) 13:405–10. doi: 10.1016/S0956-7135(01)00093-7

[7] Miyazaki I. Basic surgical techniques. J Jpn Vet Med Assoc. (1954) 7:132–5. doi: 10.12935/jvma1951.7.132

[8] Bourel C Buczinski S Desrochers A Harvey D. Comparison of two surgical site protocols for cattle in a field setting. Vet Surg. (2013) 42:223–8. doi: 10.1111/j.1532-950X.2013.01089.x, PMID: 23373589

[9] Osuna DJ Deyoung DJ Walker RL. Comparison of three skin preparation techniques in the dog part 1: experimental trial. Vet Surg. (1990) 19:14–9. doi: 10.1111/j.1532-950x.1990.tb01136.x, PMID: 2301156

[10] Ryan J Johnson JP. The equine nurse’s approach to arthroscopic surgery: part 3. Vet Nurs J. (2021) 36:13–8. doi: 10.1080/17415349.2020.1856742

[11] Fubini SL Ducharme NG Erb HN Sheils RL. A comparison in 101 dairy cows of right paralumbar fossa omentopexy and right paramedian abomasopexy for treatment of left displacement of the abomasum. Can Vet J. (1992) 33:318–24. PMID: 17424000 PMC1481252

[12] Adugna SA Kitessa JD Feyissa CT Adem SA. Review on a cesarean section in the cow: its incision approaches, relative advantage, and disadvantages. Vet Med Sci. (2022) 8:1626–31. doi: 10.1002/vms3.808, PMID: 35474614 PMC9297780

[13] Desrochers A St-Jean G Anderson DE Rogers DP Chengappa MM. Comparative evaluation of two surgical scrub preparations in cattle. Vet Surg. (1996) 25:336–41. doi: 10.1111/j.1532-950x.1996.tb01422.x, PMID: 8810024

[14] Goda H Yamaoka H Nakayama-Imaohji H Kawata H Horiuchi I Fujita Y. Microbicidal effects of weakly acidified chlorous acid water against feline calicivirus and *Clostridium difficile* spores under protein-rich conditions. PLoS One. (2017) 12:e0176718. doi: 10.1371/journal.pone.0176718, PMID: 28472060 PMC5417504

[15] Goda H Nakayama-Imaohji H Yamaoka H Tada A Nagao T Fujisawa T. Inactivation of human norovirus by chlorous acid water, a novel chlorine-based disinfectant. J Infect Chemother. (2022) 28:67–72. doi: 10.1016/j.jiac.2021.10.001, PMID: 34635450

[16] Horiuchi I Kawata H Nagao T Imaohji H Murakami K Kino Y. Antimicrobial activity and stability of weakly acidified chlorous acid water. Biocontrol Sci. (2015) 20:43–51. doi: 10.4265/bio.20.43, PMID: 25817812

[17] Ross AA Müller KM Weese JS Neufeld JD. Comprehensive skin microbiome analysis reveals the uniqueness of human skin and evidence for phylosymbiosis within the class Mammalia. Proc Natl Acad Sci USA. (2018) 115:E5786–95. doi: 10.1073/pnas.1801302115, PMID: 29871947 PMC6016819

[18] Rodrigues Hoffmann A Patterson AP Diesel A Lawhon SD Ly HJ Elkins Stephenson C. The skin microbiome in healthy and allergic dogs. PLoS One. (2014) 9:e83197. doi: 10.1371/journal.pone.0083197, PMID: 24421875 PMC3885435

[19] McBride ME Duncan WC Knox JM. The environment and the microbial ecology of human skin. Appl Environ Microbiol. (1977) 33:603–8. doi: 10.1128/aem.33.3.603-608.1977, PMID: 16345214 PMC170732

[20] Haag AF Fitzgerald JR Penadés JR. *Staphylococcus aureus* in animals. Microbiol Spectr. (2019) 7:10.1128/microbiolspec.gpp3-0060-2019. doi: 10.1128/microbiolspec.GPP3-0060-2019 PMC11257167 31124433

[21] Fey PD Olson ME. Current concepts in biofilm formation of *Staphylococcus epidermidis*. Future Microbiol. (2010) 5:917–33. doi: 10.2217/fmb.10.56, PMID: 20521936 PMC2903046

[22] Felbaum D Syed HR Snyder R McGowan JE Jha RT Nair MN. Surgical adhesive drape (IO-ban) as postoperative surgical site dressing. Cureus. (2015) 7:e394. doi: 10.7759/cureus.394, PMID: 26798570 PMC4699925

[23] Doyle AJ Saab ME McClure JT. Comparison of chlorhexidine and alcohol-based antisepsis on the paralumbar fossa in cattle. Vet Surg. (2022) 51:1191–5. doi: 10.1111/vsu.13878, PMID: 36053954

[24] Borrego L. Acute skin lesions after surgical procedures: a clinical approach. Actas Dermosifiliogr. (2013) 104:776–81. doi: 10.1016/j.ad.2013.04.001, PMID: 23791082

