# Supplementary material for: Application of chlorous acid water for disinfection of surgical site in dairy cows
Source: Front Vet Sci. 2025 Feb 26;12:1444674. doi: 10.3389/fvets.2025.1444674 (PMC11898742; doi:10.3389/fvets.2025.1444674)
Supplement: Supplementary file 1 [file Data_Sheet_1.pdf]

Supplemental figure 1. Determination of concentration of chlorous acid water for cow skin disinfection.

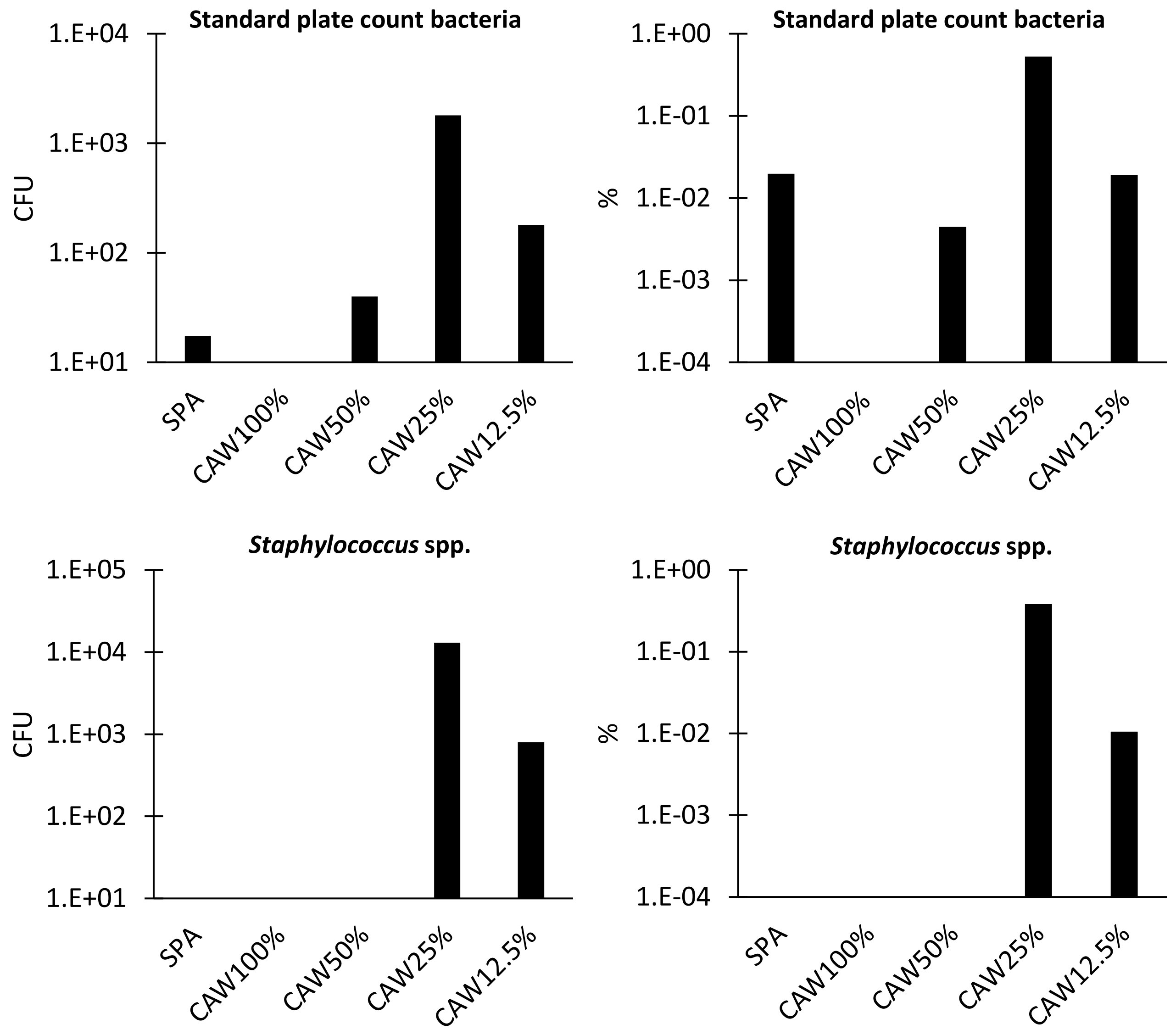

Supplemental figure 1. Determination of concentration of chlorous acid water for cow skin disinfection.

Standard disinfection protocol using scrubbing, povidone iodine, and alcohol (SPA) or modified protocol using chlorous acid water for 12.5–100% (CAW100%, CAW50%, CAW25%, CAW12.5%) are compared. Values represents after disinfection. CFU, colony forming unit/100 cm^2 skin. %, reduction rate compared to samples just after clipping.

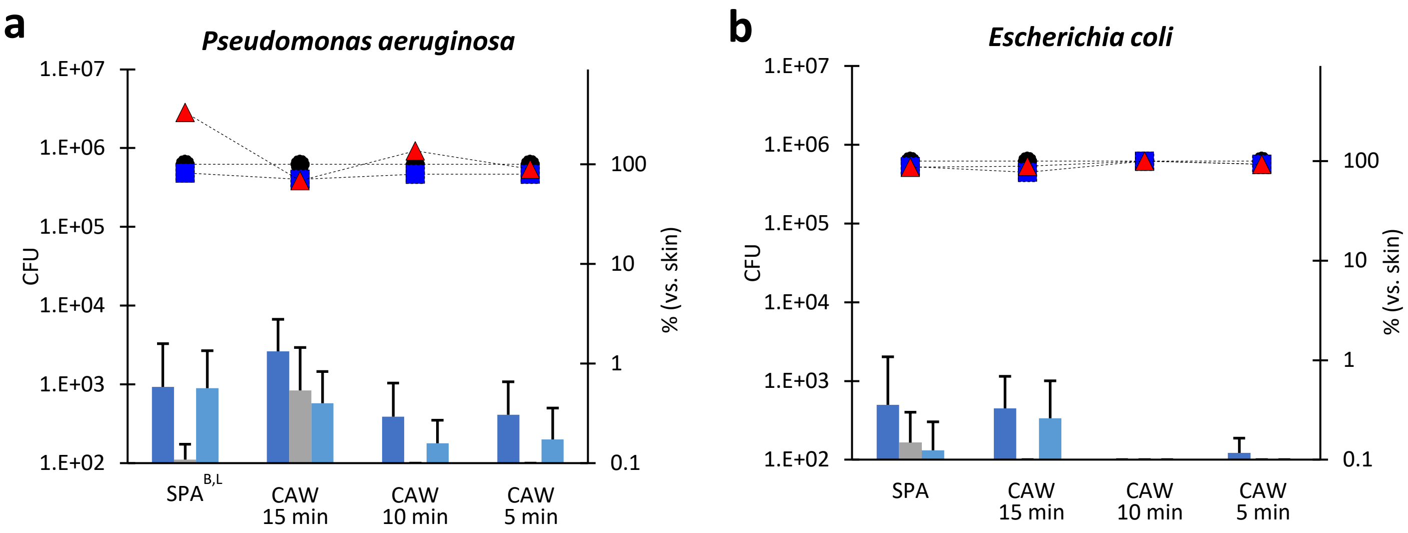

Supplemental figure 2. Bacterial flora in the skin of cows.

(a) *Pseudomonas aeruginosa*

(b) *Escherichia coli*

Standard disinfection protocol using scrubbing, povidone iodine, and alcohol (SPA) or modified protocol using chlorous acid water for 15, 10, 5 minute (CAW15, 10, 5) are compared. Bar graphs represent the colony forming unit (CFU). The line graph represents the ratio of skin samples after clipping to those after cleansing (CL) or each disinfection (DI). B, L: Indicate significances determined by the Kruskal-Wallis test ($P < 0.01$) in the bar graph and line graph, respectively. Bar graph: values = mean ± standard deviation. Line graph: values = mean.
